# Emotional reactions and baseline beliefs among people living with HIV during the second wave of the COVID-19 pandemic

**DOI:** 10.1192/j.eurpsy.2023.1721

**Published:** 2023-07-19

**Authors:** V. I. Rozhdestvenskiy, V. V. Titova, I. A. Gorkovaya, D. O. Ivanov, Y. S. Aleksandrovich

**Affiliations:** Department of Psychosomatics and Psychotherapy, Saint Petersburg State Pediatric Medical University, Saint Petersburg, Russian Federation

## Abstract

**Introduction:**

People living with HIV are at risk to develop depression, anxiety, and stress. During the pandemic, their access to medical and social care has decreased. Baseline beliefs affect the experience of mental trauma.

**Objectives:**

The study aimed to determine the levels of depression, anxiety, and stress and assess the baseline beliefs among people living with HIV. In addition, the relationship of emotional reactions to baseline beliefs was analysed.

**Methods:**

Data were collected from February 28 to July 11, 2021, using a Google form that we developed. Fifty-nine HIV-positive patients participated in the study. The DASS-21 was used to determine depression, anxiety, and stress levels, and the WAS-37 was used to examine baseline beliefs. Both questionnaires were adapted for use in Russia.

**Results:**

We found that 64 % of the respondents had no symptoms of depression, 61 % of patients reported no anxiety, and 71 % had no stress. Mean values on the scales of “Benevolence in the World” (M = 30.4±7.1) and “Justice” (M = 20.5±4.6) were below the mean normative values for the Russian population. In contrast, the scales of “Self-Image” (M = 30.1±5.4), “Luck” (M = 29.5±7.3), and “Controlling beliefs” (M = 27.1±4.4) were above the mean. Depression was associated with all types of baseline beliefs, anxiety was associated only with benevolence in the world (r_xy_ = -0.309, p < 0.05), and stress was associated with benevolence (r_xy_ = -0.281, p < 0.05) and luck (r_xy_ = -0.258, p < 0.05).

**Conclusions:**

During the COVID-19, beliefs about the world’s benevolence are associated with emotional well-being among people living with HIV.

**Disclosure of Interest:**

None Declared

